# Management of Compound Odontoma in a Pediatric Patient

**DOI:** 10.7759/cureus.77241

**Published:** 2025-01-10

**Authors:** Fouad H Althobaiti, Thekra A Almalki, Hussam I Alharthi, Muaath H Alzahrani, Omar A El Meligy

**Affiliations:** 1 Pediatric Dentistry, Ministry of Health, Taif Specialized Dental Center, Taif, SAU; 2 General Dentistry, Taif University, Taif, SAU; 3 Pediatric Dentistry, Faculty of Dentistry, King Abdulaziz University, Jeddah, SAU; 4 Pediatric Dentistry and Dental Public Health, Faculty of Dentistry, Alexandria University, Alexandria, EGY

**Keywords:** anomalies, compound odontoma, impaction, odontogenic tumor, odontoma

## Abstract

Odontomas represent the most common odontogenic tumors, typically asymptomatic and often identified incidentally during routine radiographic assessments. Although generally benign, they can impede the normal eruption of teeth. Compound odontomas, in particular, are composed of tooth-like structures and may obstruct the eruption of permanent teeth, necessitating intervention. This case report details the clinical management of a compound odontoma in an eight-year-old Saudi patient, who presented with an asymptomatic, hard swelling between the maxillary primary central incisor and canine. Clinical and radiographic evaluations revealed a radiopaque mass consistent with a compound odontoma, resulting in delayed eruption of the primary central and lateral incisors. The patient underwent surgical excision of the odontoma under general anesthesia. Careful dissection allowed the preservation of adjacent primary teeth, minimizing disruption to the dental arch and ensuring sufficient space for the normal eruption of permanent teeth. Histopathological examination confirmed the diagnosis of compound odontoma, with characteristic enamel and dentin-like structures. Postoperative recovery was uneventful, and the patient was closely monitored to observe the progression of the erupting permanent teeth. Timely identification and surgical treatment of compound odontomas are crucial to avoiding complications related to delayed tooth eruption. In this instance, the surgical removal of a compound odontoma in an eight-year-old patient preserved neighboring teeth and supported the natural eruption of permanent dentition. Regular radiographic evaluations played a key role in achieving an early diagnosis, allowing for prompt treatment and favorable oral health outcomes in pediatric care.

## Introduction

The term “odontoma” was first introduced by Paul Broca in 1867 to describe a type of tumor originating from the expansion of odontogenic tissues. Odontomas are developmental hamartomas, representing benign abnormalities composed of calcified dental tissues. These tissues result from the proliferation of fully differentiated mesenchymal and epithelial cells, which form odontoblasts and ameloblasts, contributing to the development of tooth-like structures within the tumor. According to the World Health Organization (WHO), odontomas are classified into three types: compound, complex, and ameloblastic fibro-odontomas [1].

Odontomas may develop at any age; however, they are most commonly diagnosed within the first two decades of life, often discovered incidentally during routine radiographic examinations. They typically do not show a gender predilection and primarily present as intraosseous lesions within the jaw [2]. Rare cases of odontomas occurring in soft tissues and gingiva have been reported, indicating that these lesions, though uncommon, can present in a range of anatomical sites. Compound odontomas are the more common subtype, accounting for approximately 67% of cases, while complex odontomas account for 33% of cases [3].

The etiology of odontomas remains uncertain, though several hypotheses have been proposed, including inherited genetic anomalies, inflammatory and infectious processes, local trauma during primary dentition, and genetic mutations affecting the molecular mechanisms of tooth development [4]. These factors may contribute individually or synergistically to the formation of these lesions.

The preferred treatment for odontomas is surgical excision, with a focus on complete removal to minimize the potential for recurrence. This procedure has demonstrated a high success rate with a low risk of lesion recurrence when fully excised [5].

The purpose of this case report is to present a clinical case of a compound odontoma in a pediatric patient, which was managed through surgical removal under general anesthesia (GA). This report aims to contribute to the literature on odontoma management, highlighting the importance of early diagnosis and the effective application of conservative surgical techniques in pediatric patients.

## Case presentation

Diagnosis and planning

**Figure 1:**
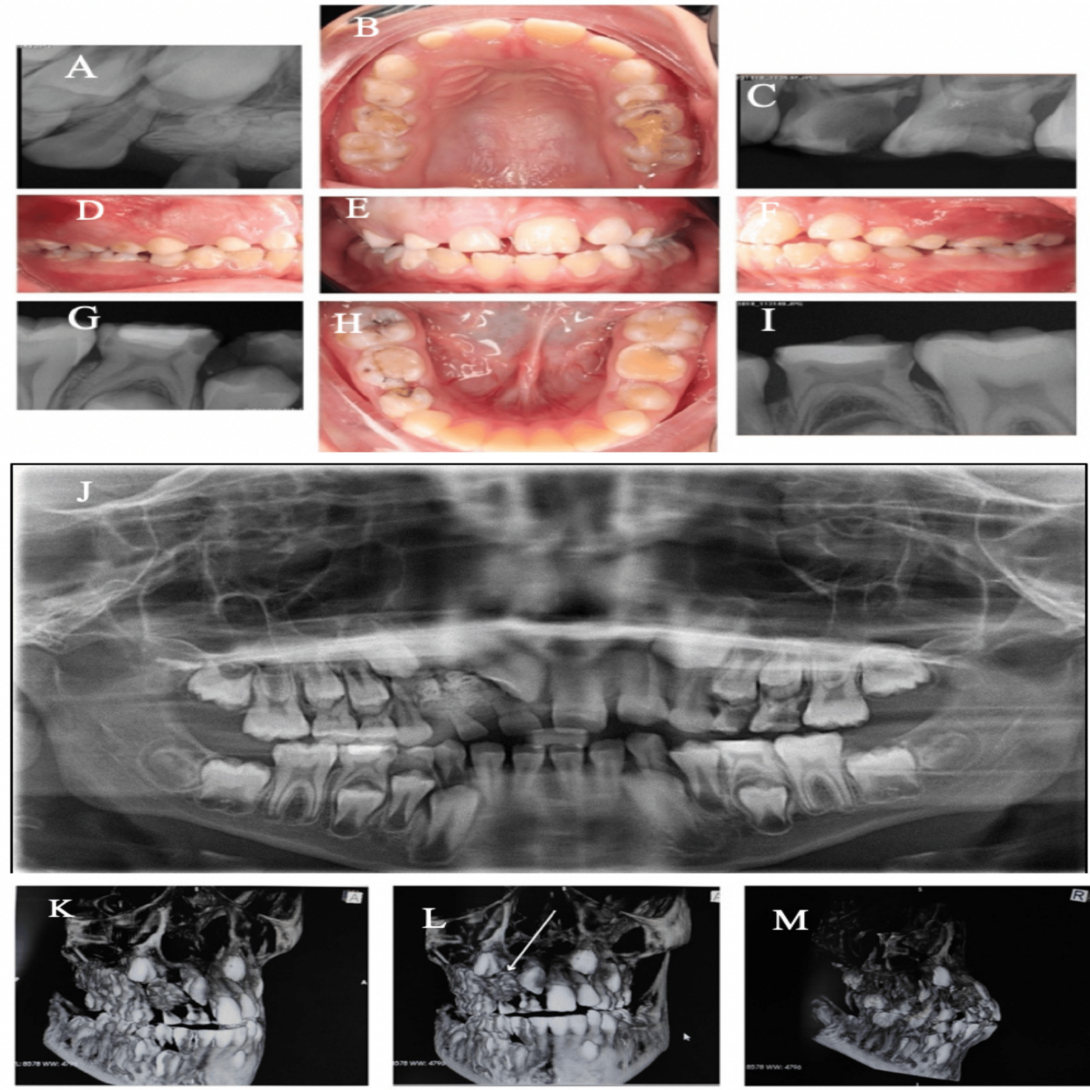
(A-I) Preoperative intraoral radiographs and clinical images illustrating localized swelling in the maxillary region, specifically between the primary central incisor and primary canine on the right side. The radiographs highlight the extent of the lesion and its relation to adjacent structures. (J) Panoramic radiograph showing a well-defined radiopaque lesion in the maxilla, consistent with the diagnosis of an odontoma. (K) CBCT sagittal plane image revealing the precise location, size, and density of the odontoma within the maxilla. (L) CBCT sagittal lateral plane image providing a lateral perspective, delineating the odontoma's proximity to surrounding anatomical structures. (M) CBCT coronal plane image demonstrating the odontoma’s spatial orientation and the degree of impaction relative to adjacent teeth and bone. CBCT, cone beam computed tomography

A healthy eight-year-old Saudi male presented to the pediatric dental clinic at the Taif Specialized Dental Center, Taif, Saudi Arabia, with his father, who reported the presence of a hard swelling related to the upper front side of his son’s jaw. Clinical examinations revealed a swelling located between the right upper primary central incisor and primary canine (Figures 1A-1I), along with delayed eruption of the upper right permanent central and lateral incisors, multiple carious teeth, defective restorations, malocclusion, generalized mild marginal biofilm-induced gingivitis, and a normal count of primary teeth with no previous trauma. A panoramic radiograph (Figure 1J), cone beam computed tomography (CBCT) (Figures 1K-1M), two horizontal bitewings, and selected periapical radiographs were obtained for the radiographic evaluation of deep carious teeth. The CBCT revealed an irregular radiopaque mass preventing the eruption of the upper right permanent central and lateral incisors, with retained primary right central and lateral incisors. The irregular mass was distopalatal to the upper right permanent central incisor and facial to the permanent lateral incisor.

Surgical procedure

**Figure 2:**
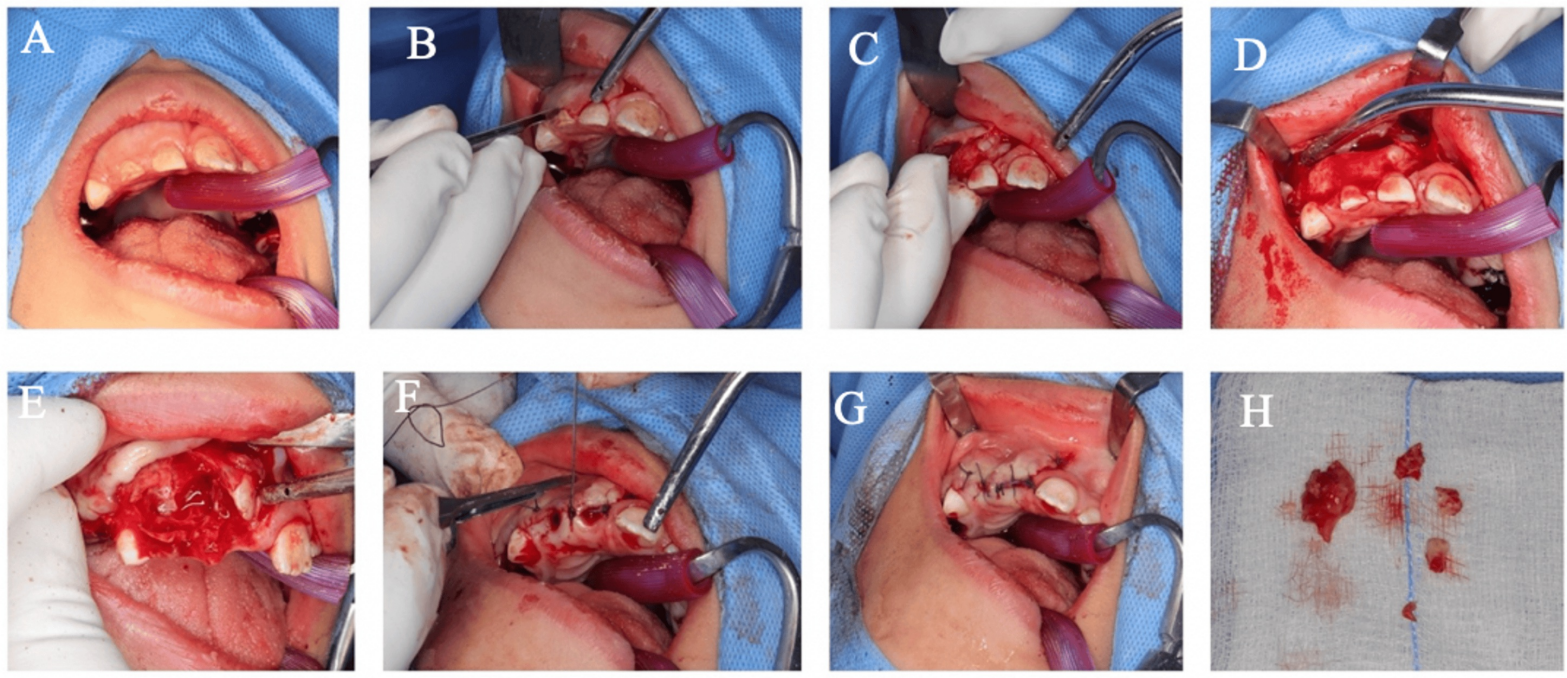
Intraoral photographs illustrating the surgical removal of the odontoma. (A) Swelling is located between the right upper primary central incisor and primary canine. (B-C) A full-thickness, mucoperiosteal, papilla-preserving flap with two releasing incisions was elevated, exposing the surgical site, and the bony prominence overlying the lesion was visualized and prepared for removal. (D-E) The outer bony surface was meticulously removed using a high-speed surgical round bur under copious saline irrigation to prevent overheating, initial exposure of the irregular odontoma mass within the maxilla, gradual isolation and mobilization of the odontoma from the surrounding bone, taking care to preserve adjacent structures, and complete removal of the odontoma along with the retained primary central and lateral incisors. (F-G) Inspection of the surgical site to confirm thorough excision, followed by closure of the flap with sutures to promote healing. (H) Odontoma along with the retained primary central and lateral incisors.

**Figure 3:**
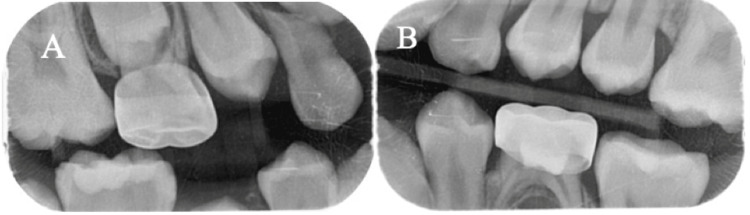
(A, B) Six-month postoperative radiographs showing proper alignment and eruption pathways of the permanent teeth, with no residual signs of the odontoma or retained primary teeth, and radiographic confirmation of healthy bone remodeling and integration around the surgical site.

**Figure 4:**
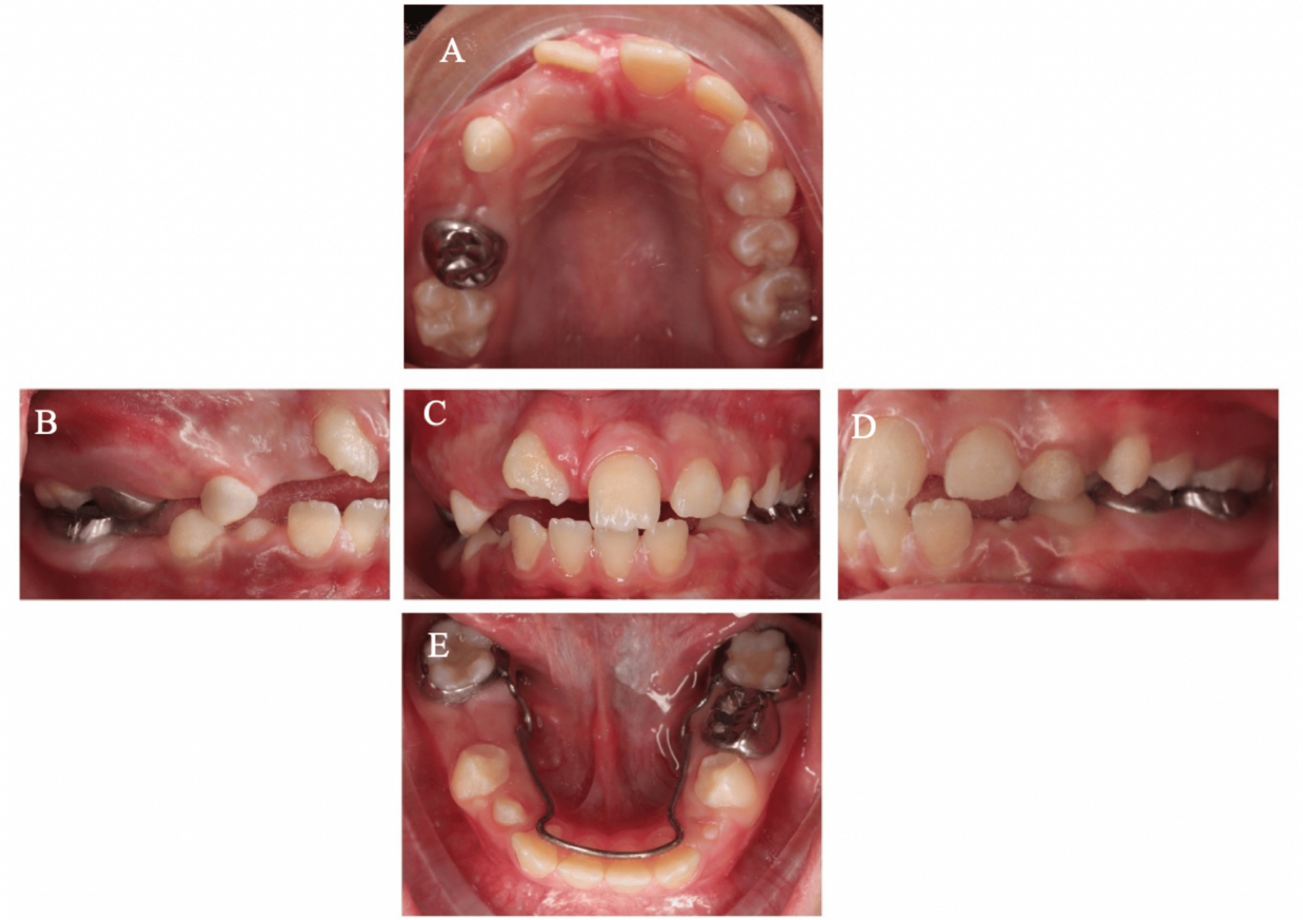
(A) Six-month postoperative views showing successful healing and significant progress in the eruption of the maxillary permanent central and lateral incisors, suggesting successful clearance of obstruction. (B-E) Clinical intraoral photograph showing the surgical region highlighting excellent soft tissue adaptation and intact restorations.

After obtaining informed consent from the parent, the treatment was performed under GA. Comprehensive dental care was provided, including the surgical removal of the odontoma, with local anesthesia administered to ensure effective pain management and hemostasis. A full-thickness, mucoperiosteal, papilla-preserving flap with two releasing incisions was performed. The bony prominence was exposed, and the outer surface was removed using a high-speed surgical round bur under cooling (Figures 2A-2H). The irregular mass was exposed and removed, along with the retained primary central and lateral incisors. A post-operative periapical radiograph was taken after the removal of the odontoma to assess the healing process. An excisional biopsy was sent to the laboratory for further analysis, which confirmed the diagnosis of an anterior maxillary compound odontoma (Table 1). A six-month follow-up revealed positive healing, significant progress in the eruption of the permanent teeth, and intact restorations (Figures 3A, 3B, 4A-4E).

**Table 1:** Vital parameters and laboratory investigation results for the specimen

Parameter	Result	Reference Range	Notes
Specimen ID	HI-23-5766	N/A	Unique identifier for the specimen
Specimen type	Multiple bony fragments	N/A	As per gross examination
Specimen size	2x2 cm	N/A	Measurement of the specimen provided
Embedding	Two cassettes	N/A	Processed with nitric acid for decalcification
Microscopic features	Normal tooth layering	N/A	Includes dentin, enamel matrix, and pulp
Histopathological diagnosis	Compound odontoma	N/A	Based on histological examination

## Discussion

Odontomas are benign hamartomatous lesions of odontogenic origin, characterized by the proliferation of fully differentiated epithelial and mesenchymal cells, leading to the formation of tooth-like structures [6]. These lesions are typically asymptomatic and often discovered incidentally during routine radiographic evaluations. The clinical manifestations of odontomas are generally associated with their impact on the eruption of permanent teeth. When odontomas obstruct the eruption pathway, they can lead to retained primary teeth, impaction, delayed eruption, or, in some cases, the complete failure of permanent teeth to erupt [7]. As odontomas account for approximately 22% of all odontogenic tumors and are the most prevalent benign tumors of odontogenic origin, arising from both epithelial and mesenchymal components, they are frequently diagnosed in pediatric patients, particularly when eruption disturbances are observed.

While complex odontomas typically develop in the posterior regions of the jaws (premolar and molar areas), compound odontomas are more commonly found in the anterior regions, especially in the maxillary incisor and canine regions. This clinical pattern aligns with the current case, where a compound odontoma was located between the right upper primary central incisor and primary canine [8]. Recognizing these regional predilections is important for early diagnosis, as the location of the lesion and its impact on adjacent teeth play a significant role in treatment planning [9].

Occlusal implications of odontomas and the importance of long-term monitoring

Odontomas can have far-reaching implications on occlusion and dental development, making long-term follow-up essential for optimal management. One of the key concerns is the potential for disruption in the normal eruption pattern of the permanent teeth. The presence of an odontoma may prevent the eruption of adjacent teeth, which can lead to misalignment or even malocclusion if not addressed. In pediatric patients, where the permanent dentition is still developing, any interruption in the eruption process can have significant consequences for future occlusal stability.

Even if the odontoma is surgically excised and the eruption of permanent teeth resumes, the patient's occlusion must be closely monitored over time. Retained primary teeth, which might remain in place due to delayed eruption of the permanent teeth, can lead to an altered bite and may influence the growth patterns of the jaw. Furthermore, once the permanent teeth begin erupting, they might be at risk for impaction or malalignment due to the space lost or disrupted by the odontoma. As such, orthodontic evaluation is crucial to assess the alignment of the emerging teeth and ensure that they follow a natural eruption trajectory.

In the case of the present patient, where the compound odontoma was excised and the adjacent primary teeth preserved, the eruption of the permanent teeth was expected to occur naturally. However, continuous monitoring is required to observe the establishment of a functional occlusion and the positioning of the permanent dentition. The close follow-up at six-month intervals, including conventional radiographic assessments, was instrumental in ensuring that no new issues arose post-surgery.

It is worth noting that while early surgical intervention can resolve many immediate issues caused by odontomas, the impact on occlusal stability may not become fully apparent until later in the eruption process. This is particularly true for patients who might experience irregular eruption patterns as they approach adolescence, a time when occlusal issues, such as crowding, open bite, or crossbite, may become more prominent. As a result, orthodontic intervention may be necessary even after the odontoma has been excised to correct any malocclusions that may have developed due to previous eruption disturbances.

Management and long-term prognosis

The management of odontomas generally involves surgical removal, which is critical in preventing further complications such as tooth loss, cyst formation, bone expansion, and delayed eruption of permanent teeth [10,11]. Enucleation and curettage are commonly used techniques to achieve complete excision of the lesion while minimizing the risk of recurrence. In the current case, the compound odontoma was successfully removed, with preservation of the adjacent primary teeth, ensuring space for the permanent teeth to erupt. Such an approach also helps in maintaining the vertical and horizontal occlusal relationships, which are crucial for overall dental function [12].

Post-surgical prognosis for odontomas is typically favorable, with a low recurrence rate when the lesion is excised completely [13]. However, the long-term management of these patients should involve a multidisciplinary approach, particularly as it pertains to the stability of occlusion. After the removal of an odontoma, it is important to assess how the permanent teeth are erupting and whether they are aligned properly. If any impaction or delayed eruption occurs, orthodontic intervention may be required to facilitate proper alignment. The use of traction and other orthodontic techniques can help guide the impacted teeth into place and prevent long-term occlusal problems [14].

In cases where complex odontomas affect the eruption of posterior teeth, the alignment of the dentition may require more extensive orthodontic treatment. Monitoring occlusion during the transition from mixed to permanent dentition is essential, as early intervention can prevent malocclusion from becoming a more significant problem later. In the present case, since the eruption of the permanent teeth progressed naturally, no additional orthodontic intervention was needed, and regular follow-up ensured that the occlusal relationships were stable.

Role of advanced imaging in diagnosis and treatment planning

The use of advanced imaging modalities, such as computed tomography (CT), plays a crucial role in the accurate diagnosis and surgical planning of odontomas. Conventional radiography remains a reliable tool for routine follow-up; however, CT imaging provides a more detailed view of the odontoma’s size, location, and relationship with adjacent structures. This three-dimensional imaging technique allows for better localization of the lesion, which is particularly useful in cases where the odontoma is complex or located in challenging anatomical areas. The ability to visualize the lesion in three dimensions enhances the precision of the surgical approach, reducing the risk of damaging adjacent teeth or bone structures [15].

## Conclusions

The early detection and surgical management of compound odontomas are essential to prevent complications from delayed tooth eruption. In this case, surgical excision of a compound odontoma in an eight-year-old patient successfully preserved adjacent teeth and facilitated the natural eruption of permanent teeth. Routine radiographic screening proved invaluable for early diagnosis, enabling timely intervention and optimal oral health outcomes in pediatric patients.
